# “Be sweet to babies”: Use of Facebook as a method of knowledge dissemination and data collection in the reduction of neonatal pain

**DOI:** 10.1002/pne2.12022

**Published:** 2020-05-02

**Authors:** Ana Claudia G. Vieira, Mariana Bueno, Denise Harrison

**Affiliations:** ^1^ School of Nursing Universidade Federal de Pelotas Pelotas Brazil; ^2^ The Hospital for Sick Children Toronto ON Canada; ^3^ Faculty of Medicine, Dentistry and Health Sciences School of Health Sciences Department of Nursing University of Melbourne Melbourne Vic. Australia

**Keywords:** breastfeeding, newborn, pain management, skin‐to‐skin contact, social media, sweet solutions

## Abstract

The Be Sweet to Babies video demonstrates the analgesic effects of breastfeeding, skin‐to‐skin care, and sweet‐tasting solutions as interventions to reduce pain during blood sampling in newborns. Although effective and safe, these strategies are implemented inconsistently in clinical settings. Given the increasing popularity of social media, there is a potential to disseminate and promote health information through it. The study aim was to  evaluate the use of Facebook as a means of disseminating the Be Sweet to Babies video in Portuguese, and to evaluate respondents’ prior knowledge, previous use of the three pain management strategies and intent to use the strategies in the future. We conducted a cross‐sectional study, using the “virtual snowball” sampling method. A Facebook webpage was created, in which the video was posted along with a brief survey. Data analyzed included number of views and visits to the page, number of views of the video, likes, dislikes, and survey responses. One year after posting, the page had 70 753 views and 2199 accesses; there were 1553 “likes”, no dislikes, and 43 positive comments. The survey was completed by 930 respondents (42% response rate based on the page access). Over two thirds of the respondents had previous knowledge about breastfeeding, skin‐to‐skin care, and sweet solutions for pain relief. After watching the video, 87% of the respondents intended to use breastfeeding or skin‐to‐skin care in the future, and 71% intended to use sweet solutions. Almost all viewers rated the video as very useful (n = 917, 99%), easy to understand (n = 926, 99%), and easy to apply in real‐life situations (n = 903, 97%). Using Facebook to deliver and evaluate an intervention is feasible, rapid in obtaining responses, low cost, and it is promising for data collection and knowledge dissemination. Further studies are warranted to evaluate the actual impact of the use of social media in practice change.

## 1 INTRODUCTION

All healthy newborns undergo at least one blood sampling for newborn screening, and preterm and sick infants undergo many more blood sampling procedures during hospitalization.^1^ Blood sampling is painful, and there is growing evidence of adverse effects of poorly treated repeated procedural pain in preterm infants.^2^ High quality synthesized evidence demonstrates analgesic effects of breastfeeding,^3^ skin‐to‐skin care,^4^ and sweet‐tasting solutions.^5^ These strategies are described as effective, safe, low cost, and feasible to implement. However, there is inconsistent use of these strategies in practice.^6^, ^7^, ^8^ To increase awareness and facilitate the implementation of evidence‐based strategies on newborn pain management, the Be Sweet to Babies team, which includes parents, clinicians, and researchers, co‐produced a 5‐minute video targeted at parents. The video demonstrates three newborns undergoing blood collection while being breastfed, held skin‐to‐skin and given sucrose (https://youtu.be/L43y0H6XEH4), and is endorsed by Baby Friendly Initiative, Ontario. The video presents user‐friendly language and is available in multiple languages.

In order to reduce gaps between what is known and what is done, researchers are increasingly using social media to promote quality information about health and to evaluate the impact of using social media as a strategy for translating knowledge into practice.^9^, ^10^, ^11^, ^12^, ^13^, ^14^ These resources are mainly targeted at families, especially parents, healthcare professionals, and the public in general. Previous studies by this team have used YouTube to disseminate the parent‐targeted and mediated Be Sweet to Babies video^12^ as well as the team's infant vaccination reduction videos.^11^ The findings of these video evaluation studies were that there were very high view rates of the videos, showing that YouTube is a useful way of sharing information to large numbers of people around the world. However, response rates to the linked viewer surveys, aimed at ascertaining previous knowledge about the pain management strategies, as well as intent to either use, or advocate for use of the pain management strategies in subsequent painful procedures, have been extremely low, only 0.24% and 1.7%.^12^ Such a low response rate was also reported by Campbell‐Yeo et al,^14^ in their study of the evaluation of a parent‐targeted video to disseminate maternal‐led comfort measures for newborns. However, a 3‐month pilot evaluation of the posting of the Portuguese language version of the Be Sweet to Babies newborn pain video, using the Facebook platform, resulted in a higher response rate based on viewers to the Facebook page. There were 1531 viewers to the page, and 709 viewers completed a linked online survey—a response rate of 46%.^13^ The Facebook platform is considered the most popular and widely used social media platform in the world when compared to platforms such as YouTube, Instagram, Twitter, and LinkedIn.^15^, ^16^, ^17^ Due to the number of connections established among those who use Facebook, there is a high possibility of reaching large numbers of people globally; in addition, it is low‐cost and a feasible method of dissemination.^15^ Based on our promising response rate after a 3‐months trial evaluation period,^13^ this current study aimed to use and evaluate the Facebook platform as a means of disseminating and evaluating the Portuguese language version of the Be Sweet to Babies video after a 12‐month period of being posted onto Facebook.

The specific aims of this study were to (a) evaluate the use of Facebook as a platform to share and evaluate the Be Sweet to Babies video; (b) evaluate viewers’ prior knowledge, viewers’ previous use of the three pain management strategies, and viewers’ intention to use the strategies in the future, and (c) evaluate acceptability, usefulness of the video.

## 2 METHODS

This was a cross‐sectional study using Facebook to host the video and the survey.

**FIGURE 1:**
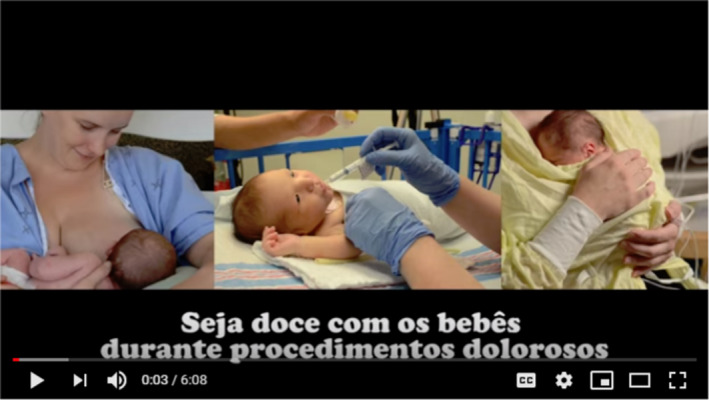
Screenshot of Portuguese be sweet to babies video

Firstly, an open‐access page on Facebook was created, supported by experts in advertising, who advised on choice of colors and layout, all inspired by the “Be Sweet to Babies” logo (https://www.facebook.com/sejadocecomosbebes). The Portuguese version of the Be Sweet to Babies (*Seja Doce com os Bebês*) video was produced and initially uploaded to YouTube in 2014, then revised and re‐uploaded in January 2016. The Portuguese Be Sweet to Babies video was produced in the audiovisual department of the Children's Hospital of Eastern Ontario (CHEO) in Ottawa, Canada (https://www.youtube.com/watch?v=nk2w‐CAVVG0). Figure 1 shows a screenshot of the Portuguese Be Sweet to Babies video.

A brief survey, developed initially in English^11^, ^18^, ^19^ and then translated to Portuguese,^19^, ^20^ was linked to the video on the Facebook page (Appendix 1). The survey included demographic questions about the respondents (parents, health professionals, researchers, and others); prior viewing of the video; prior knowledge of analgesic effects of breastfeeding, skin‐to‐skin care, and sweet solutions; intent to use or encourage the use of the pain management strategies for future newborn painful procedures; and if they would recommend the video to others (Appendix 1). Finally, respondents were asked about acceptability of the usefulness and duration of the video, and whether it was easy to understand and if the strategies were easy to apply. Participants were promoted to complete the survey immediately after watching the video by clicking on a link.

Before the page was launched, a pilot test was conducted. Twenty‐five individuals (parents of infants, researchers, and clinical professionals with expertise in the field), whose first language was Portuguese, evaluated the Facebook page in terms of functionality and clarity of the survey questions. The data collection process was also evaluated at this point. No changes were suggested or required; therefore, no modifications were made to the page or data collection processes.

Once the page was launched on September 26, 2016, invitations to access the page, to watch the video, and to complete the brief survey were distributed by e‐mail to colleagues and researchers from various states in Brazil. In addition, invitations were made through the Facebook page using “the snowball method”.^20^ Snowball sampling is an extension of convenience sampling, and results from an initial group of recruited people further recommending potential participants for the study. Those participants then recommended additional participants, and so on, thus building up like a snowball rolling down a hill. To improve awareness on the study, a summary of the results from the pilot study^13^ was published in the first author's city newspaper and in the official webpage of the affiliated University of the first author. The first author engaged with local, regional, and national press and shared the study information and the link to the Facebook page on Twitter. Finally, Facebook messages were distributed to groups of parents on Facebook, such as the Brazilian Association of Parents of Premature Babies (Prematuridade.com), seeking their social network support to collaborate and disseminate the page and the study. No paid advertisements or paid monitoring strategies were implemented.

### 2.1 Data collection

Data were collected for a 12‐month period, from September 26, 2016, to September 26, 2017. Facebook analytics in terms of number of views, geographic location where the page was viewed from, length of viewing time (to establish percentage of video viewed), number of likes, dislikes, and comments relating to the video were retrieved. The online survey was developed in Typeform, and respondents were assigned a unique ID to prevent duplicate responses. Data were exported to 18.0 SPSS software (SPSS Inc) for analysis.

### 2.2 Statistical analysis

Descriptive statistics were used to analyze Facebook analytics and survey data. The responses regarding users’ knowledge before and after watching the video were made using McNemar's test, with the significance level set at .05.^22^

Comments were reviewed by the three authors and organized by consensus into codes. Direct quotes are used to demonstrate coding of the comments.

The use of the video, including the Portuguese translation, and the study was approved by the mothers and nurses shown in the video and the Ethics Committee of the Children's Hospital of Eastern Ontario (CHEO) Research Institute, to which the last author is affiliated (REB protocol 14/108X).

## 3 RESULTS

**FIGURE 2:**
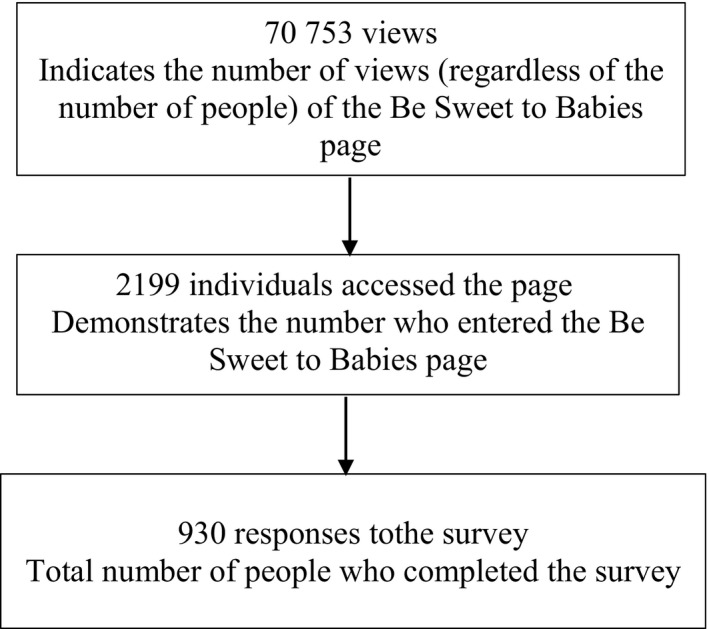
Viewer flow chart

During the 12‐month period of data collection, there were a total of 70 753 views of the Be Sweet to Babies Facebook page and a total of 2199 individuals visited the page, based on individual identifying profiles. There were 930 responses to the survey, a response rate 42.3% based on the 2199 individuals who accessed the page (Figure 2).

As summarized in Table 1, most participants (n = 763; 82%) used a smartphone to access the page and complete the survey; 139 (15%) used computers or laptops, and 28 (3%) used tablets. Over two thirds of the respondents identified themselves as healthcare professionals (n = 633, 68%) while 223 (24%) identified themselves as parents or guardians; finally, 25 (3%) were identified as researchers. Just over half of the respondents (510; 55%) had not viewed the video before.

**TABLE 1:** Respondents’ characteristics and demographics (n = 930)

Variables	N	%
Viewer identification		
Health professionals	633	68
Parents or guardians	223	24
Researchers	25	3
Others	49	5
Previous viewing		
Not viewed before	510	55
Others (viewed previously via other methods)	291	31
YouTube	89	10
University course	21	2
Conference	12	1
Online conference	7	1
Viewing method		
Smartphone	763	82
Computer or laptop	139	15
Tablet	28	3

Time (minutes) taken to complete the survey	Mean
Smartphone	06:14
Computer or laptop	05:02
Tablet	03:17

The majority of the respondents rated the video as very useful (n = 917, 99%), easy to understand (n = 926, 99%), and easy to apply in real‐life situations (n = 903, 97%). After watching the video, 928 respondents (99.8%) intended to use or advocate for the use of all three pain management strategies and only two respondents answered they would not recommend the video to others. After watching the video, respondents stated they wished to use breastfeeding or skin‐to‐skin contact (n = 807, 87%, and n = 808, 87%, respectively), while 659 (71%) intended to use sucrose. Table 2 presents a summary of the survey results.

**TABLE 2:** Previous knowledge and intention to use one of the three pain management strategies following viewing of The Be Sweet to Babies video

Pain management strategies	Before the video^a^ (n = 930) n (%)	After the video^b^ (n = 930) n (%)	*P*
Breastfeeding, n (%)			
Yes	702 (76)	807 (87)	<.001
No	228 (25)	123 (13)	
Skin‐to‐skin contact, n (%)			
Yes	679 (73)	808 (87)	<.001
No	251 (27)	122 (13)	
Sucrose, n (%)			
Yes	593 (64)	659 (71)	<.001
No	337 (36)	271 (29)	

Note

Data presented about relative and absolute frequency; *p* value refers to the McNemar's test.

^a^ Before viewing the Be Sweet to Babies video, did you know that breastfeeding, skin‐to‐skin contact, or sucrose is effective at reducing pain during procedures for babies?

^b^ After viewing the Be Sweet to Babies video, do you intend to use or advocate for one of the three pain management strategies?

The majority of viewers agreed the duration of the video was ideal (n = 805, 86%) with only 118 (13%) reporting the video as too long. On the Facebook page, the video received 1553 “likes” and 43 positive comments. The comments were organized into three categories: usefulness, general praise, and recommendations. For example, the comment “The study brought me simple practices with scientific foundations that really work! My rating is excellent! I will share and put into my professional practice” was coded into usefulness. General praise included comments such as “Exemplary work and the most beautiful of all is to see the care and care that each one has with the babies”. The comment “Super recommend breastfeeding while the baby takes the vaccine…” was coded into Recommendations. Key comments which best illustrate the three codes were translated by the first author and second author and summarized in Table 3.

**TABLE 3:** Coding and example comments

Usefulness	General praise	Recommendations
The study brought me simple practices with scientific foundations that really work! We can greatly help reduce the discomfort of our babies Relieving pain in "usually" painful procedures is a challenge for us health professionals, especially for babies Congratulations. Simple methods that make the difference I’m a mother for the second time, and I posted on my Facebook that I was devastated because of the first dose of the vaccines on my baby's thighs…Hence a friend, who is a nurse told me about the study of breastfeeding while my baby received doses of vaccine…And incredible, it worked! My baby did not feel any more pain in the applications of the vaccines, much less reactions	I learnt about the work by Dr Vieira today at CBCENF in Rio de Janeiro and I simply loved it Relieving pain in "usually" painful procedures is a challenge for us health professionals, especially for babies Beautiful project, congratulations… Very good! Exemplary work and the most beautiful of all is to see the care and care that each one has with the babies Congratulations and nice to see that there are still people like this A wonderful study.	My rating is Excellent! I will share with my colleagues and put into my professional practice…Very good Super recommend breastfeeding while the baby takes the vaccine… loved it! Relieving pain in “usually” painful procedure is a challenge for us health professionals, even more so when it comes to babies. A necessary initiative for the physical and mental health of infants.

There were no dislikes or negative comments posted into the Facebook page.

## 4 DISCUSSION

After 1 year, the Portuguese language Be Sweet to Babies video, posted on the Facebook social media platform, had a large number of views (70 753) and the Be Sweet to Babies Facebook page was accessed 2199 times by healthcare professionals, parents, and researchers across 45 municipalities from different states of Brazil. There were 930 surveys completed (42% of individuals accessing the page), which highlights the potential value of Facebook for disseminating information, increasing awareness of evidence, and also for data collection.

Social media platforms are being used increasingly by healthcare professionals and researchers to share information about health and research evidence.^21^ This study builds on other studies evaluating the potential use and effectiveness of social media platforms in disseminating synthesized and distilled best evidence in the form of brief videos. For example, a brief video of 1 minute 35 seconds duration, targeted at parents demonstrating the analgesic effects of breastfeeding and sucrose during vaccination, was evaluated following a 12‐month period of being posted onto YouTube.^11^ In the 12‐month time period, the video was viewed almost 65 500 times, and similar to this current study, received large numbers of “likes.” The content of comments was, however, different, with the vaccination video attracting anti‐vaccination comments and positive comments about the videos. In another similar study, Harrison et al^12^ evaluated the reach and dissemination of the English version of the Be Sweet to Babies newborn pain video. After 12 months of being posted onto YouTube, the video had nearly 11 000 views, a large number of likes, but only three comments. Similarly, another newborn pain video posted onto YouTube, which also targeted parents, was evaluated after an 18‐month period postrelease.^14^ This video had a larger number of views, nearly 158 000, 87 likes and 73 comments in total. These large numbers of views highlight the great potential for using social media platforms to share information.

Although these results are promising in terms of the extremely wide reach of the information posted onto social media, using these platforms to collect participants’ data in order to evaluate acceptability and effectiveness of the information shared has been less successful to date. For the three studies discussed above,^11^, ^12^, ^14^ viewer surveys were linked from YouTube to collect information on previous knowledge of the evidence; previous use of the recommended pain management strategies; acceptability of the videos, intent to use the recommended pain management strategies in future painful procedures; and intent to recommend the video to others. For the infant vaccination video,^12^ only 156 of the 65 000 viewers completed the survey, a response rate of only 0.24%. For the two other newborn pain videos, survey response rates based on the viewer rates were also very low, 0.13% and 1.7%, respectively.^12^, ^14^ This large number of views overall, yet with extremely low response rates when viewer surveys were linked from the YouTube video itself, highlights the benefits of sharing health information online, but there are still challenges in rigorously evaluating the videos and their potential effectiveness in changing and improving practices. However, in this current study, the video was not viewed on YouTube; instead, the video was inserted on the Facebook page itself, to prevent viewers from being redirected to YouTube. Based on the 2000 viewers that accessed the Facebook page, 930 completed the survey, giving a much higher response rate of 42% with no dislikes or negative comments. However, as stated in the results, the number of people viewing the page, without accessing the actual page, was much higher, at 70 753 page views. If this is taken as the denominator to calculate response rates to the survey, that brings response rates down to similar rates as the other studies, of 1.3% only. As above, this highlights ongoing challenges with using social media platforms to evaluate any process or clinical outcomes.

Based on the completed surveys for this current study, the two other newborn pain studies,^12^, ^14^ and the infant vaccination video study,^11^ the videos were persuasive, and most respondents reported they intended to use the pain management strategies demonstrated in the videos in the future. The positive responses from the majority of viewers are in line with another study of the Portuguese Be Sweet to Babies video, which was evaluated with parents of infants in a neonatal intensive care unit in Brazil.^22^ Before being shown the video, most parents reported they did not know about breastfeeding, skin‐to‐skin care, or sweet solutions for reducing newborn pain during painful procedures. However, after viewing the video, all 100 of the parents reported they would use one of the three pain management strategies. Overall, although these studies indicate the video as a persuasive tool, further research is still required to ascertain whether the strategies are actually implemented in clinical practice.

Information shared on social media may create awareness of best practices and may provide motivation to change but it is not yet known whether the information directly changes behaviors. Although this process represents an opportunity to share common interest or engage parents, it is a challenge to present information and build credibility.^23^ In addition to that, further challenges include identifying how patients and families perceive the use of social media in research, how they prefer to be involved, and whether different tools or approaches are more effective for different questions, in order to establish guidance/a model or standard on using social media in different health contexts.^23^

In another study, a video demonstrating pain management strategies recommended during infant vaccination, which included breastfeeding, sucrose, and topical anesthetics, was shown at prenatal classes.^24^ Of the 88 mothers included in the video group, only one third used one of the three strategies during their infant's 2‐month vaccination, and another 16% attempted to breastfeed, but were unsuccessful in actually carrying this out. The reasons given were that the clinicians did not approve or allow this to occur.^24^ This is despite the high quality synthesized evidence of effectiveness of breastfeeding during infant vaccination^25^ and the World Health Organization recommendations to breastfeed during vaccinations.^26^ Understanding and addressing such clinician related factors will be important in future studies aiming to ascertain effectiveness of interventions aimed to improve practices. Nurses’ and other HCPs’ ongoing use of social media platforms to widely disseminate best evidence and to engage the public has been recommended.^27^ Since the popularity of the use of social media has changed the way of obtaining information, HCPs and researchers need to update themselves and understand how to use them in order to provide quality information.^28^ As researchers and HCPs, it is recognized that there is an obligation to widely share research findings, especially with end users such as families.^27^ Increasingly, researchers are working at increasing access for parents, to pediatric pain‐related knowledge, as highlighted in a recently published systematic review which included 12 studies of programs aimed at sharing and implementing evidence‐based pain related knowledge to parents.^29^ In this current study, widely sharing best newborn pain management evidence in Brazil, by means of the parent‐targeted Portuguese language Be Sweet to Babies video, posted on the Facebook social media platform, aimed to do just that, with the ultimate aim of translating the knowledge into practice.

### 4.1 Strengths and limitations

This study of the use of Facebook to disseminate the parent‐targeted Be Sweet to Babies video and evaluating respondents’ perceptions of the video resulted in large numbers of views, visits to the page, and a good survey response rate. The response rate of 42% of viewers who accessed the Facebook page is much higher than previous studies evaluating the video when posted onto YouTube. In addition, using Facebook as the social media platform for sharing information is at a low cost when compared to administering surveys in person, or by post, and can reach large numbers of people in diverse geographic regions. However, as per previous studies by this team and others, positive findings regarding potential implementation of the evidence‐based information shared using social media platforms can only inform intention to use the information in the future, and not the actual impact, and whether the recommended practices are actually put into practice. Furthermore, it is not known if viewers completed the survey more than once. Using more traditional research methods to evaluate whether the knowledge translation products, such as the Be Sweet to Babies videos, in conjunction with dissemination through social media, are warranted to evaluate actual practice change in the clinical area. In addition, although using social media results in widespread sharing of information, using Facebook does involve time and commitment in developing and monitoring pages, and keeping information up to date and targeted at the intended audience.

## 5 CONCLUSIONS

The brief parent‐targeted Portuguese language Be Sweet to Babies video demonstrating use of breastfeeding, skin‐to‐skin care, and sweet‐tasting solutions for newborns during painful procedures, and shared through the Facebook page, had a large number of views in a 1‐year period, and based on access to the page, the survey had a good response rate of 42%. The video was considered useful and persuasive in terms of future intent to use the pain management strategies. Widely disseminating the video to the public, and using Facebook to collect viewers’ data, was feasible, and a quick and inexpensive method to obtain responses. Although the results are positive, further studies evaluating whether the viewing of the video actually changes clinical practices in terms of neonatal pain management strategies are warranted.

## Appendix 1 Survey questions

**Table:**

1. Are you: Parent or guardian Health Care Provider Researcher Other
2. Had you seen the *Be Sweet to Babies* video before today? Yes No If so, where? YouTube Conference University course Webinar Other
3. Before viewing the *Be Sweet to Babies* video, did you know that: Breastfeeding is effective at reducing pain during procedures for babies? Yes No Skin to skin care is effective at reducing pain during procedures for babies? Yes No Sugar water (sucrose) is effective at reducing pain during procedures for babies? Yes No
4. After viewing the *Be Sweet to Babies* video, do you intend to use or advocate for one of the three pain management strategies? Yes No If so, which? Breastfeeding Skin‐to‐Skin Sucrose
5. Would you recommend the *Be Sweet to Babies* video to other parents? Yes No Unsure
6. Was this video: Helpful or beneficial for you? Yes No Easy to understand? Yes No Easy to apply in real life situations? Yes No
7. What did you think about the length of the *Be Sweet to Babies* video? Just right Too long Too short
